# Supplementary material for: The Perth Empathy Scale: Psychometric Properties of the Polish Version and Its Mental Health Correlates
Source: Eur J Investig Health Psychol Educ. 2023 Nov 10;13(11):2615–29. doi: 10.3390/ejihpe13110182 (PMC10670358; doi:10.3390/ejihpe13110182)
Supplement: Supplementary file 1 [file ejihpe-13-00182-s001.zip › ejihpe-2639624-supplementary.pdf]

Table S1. The Polish version of the Perth Empathy Scale

Skala empatii Perth (polska wersja: Larionow i Preece, 2023)

The Perth Empathy Scale (PES; Brett et al., 2022)

Instrukcja. Ten kwestionariusz pyta o to, jak łatwo rozpoznajesz i doświadczasz emocji innych. Proszę oceń następujące twierdzenia przy użyciu podanej skali. Zakreśl jedną odpowiedź dla każdego twierdzenia.

	Twierdzenia	Prawie nigdy	Rzadko	Czasami	Często	Prawie zawsze
1	Widząc lub słysząc kogoś, wiem, czy czuje się smutny.	1	2	3	4	5
2	Kiedy widzę lub słyszę kogoś, kto czuje się smutny, to sprawia, że ja też czuję się smutna/y.	1	2	3	4	5
3	Widząc lub słysząc kogoś, wiem, czy czuje się szczęśliwy.	1	2	3	4	5
4	Kiedy widzę lub słyszę kogoś, kto czuje się szczęśliwy, to sprawia, że ja też czuję się szczęśliwa/y.	1	2	3	4	5
5	Widząc lub słysząc kogoś, wiem, czy czuje się zły.	1	2	3	4	5
6	Kiedy widzę lub słyszę kogoś, kto czuje się zły, to sprawia, że ja też czuję się zła/y.	1	2	3	4	5
7	Widząc lub słysząc kogoś, wiem, czy czuje się rozbawiony.	1	2	3	4	5
8	Kiedy widzę lub słyszę kogoś, kto czuje się rozbawiony, to sprawia, że ja też czuję się rozbawiona/y.	1	2	3	4	5
9	Widząc lub słysząc kogoś, wiem, czy czuje się przestraszony.	1	2	3	4	5
10	Kiedy widzę lub słyszę kogoś, kto czuje się przestraszony, to sprawia, że ja też czuję się przestraszona/y.	1	2	3	4	5
11	Widząc lub słysząc kogoś, wiem, czy czuje się spokojny.	1	2	3	4	5
12	Kiedy widzę lub słyszę kogoś, kto czuje się spokojny, to sprawia, że ja też czuję się spokojna/y.	1	2	3	4	5
13	Widząc lub słysząc kogoś, wiem, czy czuje się zniesmaczony.	1	2	3	4	5
14	Kiedy widzę lub słyszę kogoś, kto czuje się zniesmaczony, to sprawia, że ja też czuję się zniesmaczona/y.	1	2	3	4	5
15	Widząc lub słysząc kogoś, wiem, czy czuje się pełen zapału.	1	2	3	4	5
16	Kiedy widzę lub słyszę kogoś, kto czuje się pełen zapału, to sprawia, że ja też czuję się pełna/pełen zapału.	1	2	3	4	5
17	Widząc lub słysząc kogoś, wiem, czy czuje się zakłopotany.	1	2	3	4	5
18	Kiedy widzę lub słyszę kogoś, kto czuje się zakłopotany, to sprawia, że ja też czuję się zakłopotana/y.	1	2	3	4	5
19	Widząc lub słysząc kogoś, wiem, czy czuje się dumny.	1	2	3	4	5
20	Kiedy widzę lub słyszę kogoś, kto czuje się dumny, to sprawia, że ja też czuję się dumna/y.	1	2	3	4	5

Obliczanie wyników w Skali empatii Perth

Empatia to wielowymiarowy konstrukt składający się z dwóch komponentów: empatii poznawczej i empatii afektywnej. Empatia poznawcza odnosi się do zdolności rozpoznawania emocji innych, podczas gdy empatia afektywna odnosi się do zdolności wczuwania się w emocje oraz doświadczania emocji innych. Innymi słowy, ludzie o wysokim poziomie empatii mogą z łatwością rozpoznawać emocje innych i doświadczać tych emocji.

Skala empatii Perth to 20-pozycyjny samoopisowy kwestionariusz, który służy do oceny zarówno poznawczych, jak i afektywnych komponentów empatii w stosunku do negatywnych i pozytywnych emocji oddzielnie. Z pomiaru można uzyskać wyniki dla czterech podskal i trzy wyniki złożone, przy czym wyższe wyniki wskazują na wyższy poziom empatii. Łączny wynik (ogólna empatia) może być obliczony poprzez zsumowanie wszystkich pozycji.

Poniższa tabela opisuje każdą podskalę Skali empatii Perth i wyniki złożone oraz sposób ich obliczania.

Podskale/wyniki złożone	Jak obliczyć?	Co mierzy?
**Wyniki podskal**		
Empatia Negatywno-Poznawcza	Suma pozycji 1, 5, 9, 13, 17	Umiejętność rozpoznawania negatywnych emocji innych
Empatia Pozytywno-Poznawcza	Suma pozycji 3, 7, 11, 15, 19	Umiejętność rozpoznawania pozytywnych emocji innych
Empatia Negatywno-Afektywna	Suma pozycji 2, 6, 10, 14, 18	Umiejętność wczuwania się w negatywne emocje innych
Empatia Pozytywno-Afektywna	Suma pozycji 4, 8, 12, 16, 20	Umiejętność wczuwania się w pozytywne emocje innych
**Wyniki złożone**		
Ogólna Empatia Poznawcza	Suma wyników podskal Empatia Negatywno-Poznawcza i Empatia Pozytywno-Poznawcza	Umiejętność rozpoznawania emocji innych (negatywnych i pozytywnych)
Ogólna Empatia Afektywna	Suma wyników podskal Empatia Negatywno-Afektywna i Empatia Pozytywno-Afektywna	Umiejętność wczuwania się w emocje innych (negatywne i pozytywne)
Ogólna Empatia (wynik ogólny)	Suma wszystkich pozycji	Ogólna empatia; umiejętność rozpoznawania i wczuwania się w emocje innych (negatywne i pozytywne)
